# Supplementary material for: TFP Bioeconomy Impact post Covid-19 on the agricultural economy
Source: PLoS One. 2023 Nov 17;18(11):e0288885. doi: 10.1371/journal.pone.0288885 (PMC10656026; doi:10.1371/journal.pone.0288885)
Supplement: Attachment — Submitted filename: review opinions.docx [file pone.0288885.s001.docx]

The author used panel date to investigate the impact of Covid-19 on bio-economy from six regions in the world, I think the aim of this paper is worth looking into, however, this paper needs tremendous polishing before it could meet publication requirement.

1. Language needs to be polished by an native English professional. This paper reads ambiguously and redundantly.
2. In the second line of the introduction, the author had put “Some authors have pointed out” without citing the work you were referring to.
3. In the forth paragraph of the introduction, the authors put “so the contribution of this study is focused on measuring the change in TFP in six regions of the world. Part of the research efforts” . From my perspective, the authors were trying to put forward the significance of the paper, however, you did not explain clearly why and how this method and this outcome would serve these means.
4. By the end of this paragraph, there was an extra “That”
5. The last paragraph of the introduction, the authors seemed to left out the number this paper was divided into, therefore this paragraph was a little confusing. The authors should treat this submission more seriously and avoid such mistakes.
6. I would suggest the authors reorganize the introduction part, explain clearly the background of the research, the methods you were using and why is was appropriate, the aim and objectives of this paper, and what outcome and achievement you were expecting from this paper.
7. I was confused by the way the authors arranged this paper, as you used Roman numbers for the introduction and literature review section but there was no numbering for total factor productivity. I suggest you rearrange each section and make it clear.
8. Even though the authors have listed many related papers in the literature review section, the discussion was absent as you went directly from literature review to total factor productivity. Literature review section was for you to list out related works and comment on the research background, then it leads to the research blank that this paper should be fulfilling. Without the discussion, the literature review is not complete.
9. Methods and data should be merged into one big section, I suggestion the authors state clearly why this method was employed first, then construct your model, then explain the data source. I don’t understand why controlled variables were not explain along with the model you constructed.
10. For statistics, I suggest the authors put a table of inputs and outputs and then list the statistics for discussion.
11. The authors had discussed the results from your data, however, you did not form an discussion with existing literatures, I suggest you add a “discussion” section to compare your research findings with existing research and discussion the significance and difference in your findings.
12. The aims of this paper is meaningful, therefore I look forward to see the finely polished version of this paper.
