# Supplementary material for: TFP Bioeconomy Impact post Covid-19 on the agricultural economy
Source: PLoS One. 2023 Nov 17;18(11):e0288885. doi: 10.1371/journal.pone.0288885 (PMC10656026; doi:10.1371/journal.pone.0288885)
Supplement: Attachment — Submitted filename: review opinions.docx [file pone.0288885.s002.docx]

6/10/2023

Noé Aguilar-Rivera

Editor Academico

Dear editor thanks for all observations

Dear Editor, thank you for all your observations that surely contribute to improving the quality of the research. Below I present in detail each of the improvements incorporated.
All the change made go in yellow color.

The author used panel data to investigate the impact of Covid-19 on the bio-economy from six regions in the world, I think the aim of this paper is worth looking into, however, this paper needs tremendous polishing before it could meet publication requirements.

I appreciate your comments. The panel data is an essential condition for the Malmquist indices. I agree to make the results more visible in the face of regionalization and the measurement of productivity and efficiency that after Covid the governments justified with their respective economic policies.

1. Language needs to be polished by a native English professional. This paper reads ambiguously and redundantly.

A review was done with a native English colleague, I hope your valuable observations in this regard are improved.

2. In the second line of the introduction, the author had put “Some authors have pointed out” without citing the work you were referring to.

It was added.

3. In the fourth paragraph of the introduction, the authors put “so the contribution of this study is focused on measuring the change in TFP in six regions of the world. Part of the research efforts”. From my perspective, the authors were trying to put forward the significance of the paper, however, you did not explain clearly why and how this method and this outcome would serve these means.

Thanks for this observation. It was eliminate and added in the fifth paragraph, I saw necessary separate in the next paragraph. This paragraph was reword.

4. By the end of this paragraph, there was an extra “That”

It was eliminate

5. In The last paragraph of the introduction, the authors seemed to left out the number this paper was divided into, therefore this paragraph was a little confusing. The authors should treat this submission more seriously and avoid such mistakes.

Thanks for this observation. It was correct.

6. I would suggest the authors reorganize the introduction part, explain clearly the background of the research, the methods you were using and why is was appropriate, the aim and objectives of this paper, and what outcome and achievement you were expecting from this paper.

Thanks for this observation. It was added the table3 that resume the TFP used in bio based economic. The section was re organized and clarify the background, aim, contribution and objective.

7. I was confused by the way the authors arranged this paper, as you used Roman numbers for the introduction and literature review section but there was no numbering for total factor productivity. I suggest you rearrange each section and make it clear.

My sincerest apologies for that. That was duly numbered both by section and by subsections.

8. Even though the authors have listed many related papers in the literature review section, the discussion was absent as you went directly from literature review to total factor productivity. Literature review section was for you to list out related works and comment on the research background, then it leads to the research blank that this paper should be fulfilling. Without the discussion, the literature review is not complete.

I also apologize for this observation. The literature review focused on two components: one was the bioeconomy and the other was total factor productivity. One is the theoretical approach towards which we want to measure and the other is the tool that will measure it. Make an effort to bring these two components together in the discussion you recommend. Thanks for this observation.

9. Methods and data should be merged into one big section, I suggestion the authors state clearly why this method was employed first, then construct your model, then explain the data source. I don’t understand why controlled variables were not explain along with the model you constructed.

The model was contructed and explained.

It was made. 3. 1 3.1 Control Variables and Panel Data Sources

10. For statistics, I suggest the authors put a table of inputs and outputs and then list the statistics for discussion.

It was added.

11. The authors had discussed the results from your data, however, you did not form an discussion with existing literatures, I suggest you add a “discussion” section to compare your research findings with existing research and discussion the significance and difference in your findings.

Yes it was made. It was added.

12. The aims of this paper is meaningful, therefore I look forward to see the finely polished version of this paper.

Thank you for your comment.
