# Supplementary material for: TFP Bioeconomy Impact post Covid-19 on the agricultural economy
Source: PLoS One. 2023 Nov 17;18(11):e0288885. doi: 10.1371/journal.pone.0288885 (PMC10656026; doi:10.1371/journal.pone.0288885)
Supplement: Attachment — Submitted filename: Rebuttal Letter.docx [file pone.0288885.s003.docx]

2023/07/04

Noé Aguilar-Rivera

Academic Editor

PLOS ONE

Dear academic editor, thank you for your valuable management in this editorial process. Below I present the rebuttal letter indicating each of the improvements to the reviewers' observations.

Comments to the Author

1. If the authors have adequately addressed your comments raised in a previous round of review and you feel that this manuscript is now acceptable for publication, you may indicate that here to bypass the “Comments to the Author” section, enter your conflict of interest statement in the “Confidential to Editor” section, and submit your "Accept" recommendation.

Reviewer #1: (No Response)

Reviewer #2: All comments have been addressed

2. Is the manuscript technically sound, and do the data support the conclusions?

The manuscript must describe a technically sound piece of scientific research with data that supports the conclusions. Experiments must have been conducted rigorously, with appropriate controls, replication, and sample sizes. The conclusions must be drawn appropriately based on the data presented.

Reviewer #1: Yes

Reviewer #2: Partly

3. Has the statistical analysis been performed appropriately and rigorously?

Reviewer #1: Yes

Reviewer #2: Yes

4. Have the authors made all data underlying the findings in their manuscript fully available?

The PLOS Data policy requires authors to make all data underlying the findings described in their manuscript fully available without restriction, with rare exception (please refer to the Data Availability Statement in the manuscript PDF file). The data should be provided as part of the manuscript or its supporting information, or deposited to a public repository. For example, in addition to summary statistics, the data points behind means, medians and variance measures should be available. If there are restrictions on publicly sharing data—e.g. participant privacy or use of data from a third party—those must be specified.

Reviewer #1: Yes

Reviewer #2: Yes

5. Is the manuscript presented in an intelligible fashion and written in standard English?

PLOS ONE does not copyedit accepted manuscripts, so the language in submitted articles must be clear, correct, and unambiguous. Any typographical or grammatical errors should be corrected at revision, so please note any specific errors here.

Reviewer #1: Yes

Reviewer #2: Yes

6. Review Comments to the Author

Please use the space provided to explain your answers to the questions above. You may also include additional comments for the author, including concerns about dual publication, research ethics, or publication ethics. (Please upload your review as an attachment if it exceeds 20,000 characters)

Reviewer #1: The language has been improved significantly, as well as the quality of the paper. However, there are still some shortcoming I think the authors should work on：

1. The literature review section still lacks review and discussion.

I appreciate the reviewer's comments.Corrections were made, marked in yellow. A paragraph was added at the beginning of section 2.1 and at the end of section 2.3, a paragraph was added summarizing the contributions of research on the subject of bioeconomy, and the Malmquist indices tool to measure efficiency and productivity. of the bioeconomy during and post covid. The emptiness is made clear and hence the contribution of this work.

2. You need to discuss the descriptive statistics before starting analyzing.

We appreciate the reviewer's comments. The discussion of Table 1 moved to the results and discussion section before starting the analysis. It Added the discussion.

3. Form a discussion with current literature by comparing your results to other research before you draw the conclusions.

I look forward to see the three sections added before this paper goes to publication.

Thanks for your observation, the tree section were added.

Reviewer #2: Thank you for the new version of the paper which has beenmeaningfully improved. I still have probems with the literature review, which seems to me quite random and it is not very clear how and to what extent your work contribute to that strand of literature.

Dear thanks for these observations. Two paragraph has added with the intention of combine the bioeconomy with the índices Malmquist tool. The use of Indices Malmquist as measure tool of the Bioeconomy is a contribution study with controlled variables to level regional decreased or were not productive or technically efficient.

I find fig. 4 and fig. 5 a bit useless and fig. 6 quite unreadable.

Regarding the fig. 4 is for indicating region Covid affectation. The fig 4 is for indicating region of the study both suggested by back reviewer. Fig 5 is with the standard as journal ask.

Regarding the Fig 6 is the better resolution on The VOSviewer software (RRID: SCR_023516) it also, was suggested by other back reviewer.

I also suggest to improve the conclusions to highlight your original contribution and addition to the existing literature status quo.

Thanks for this information and observation. It was added and improved.

7. PLOS authors have the option to publish the peer review history of their article (what does this mean?). If published, this will include your full peer review and any attached files.

If you choose “no”, your identity will remain anonymous but your review may still be made public.

Do you want your identity to be public for this peer review? For information about this choice, including consent withdrawal, please see our Privacy Policy.

Reviewer #1: No

Reviewer #2: No

Kind regards,

C.A. Zuniga-Gonzalez

Researcher

UNAN Leon
